# Unexpected Repercussions of the COVID-19 Pandemic on Total Hip Arthroplasty with Cemented Hip Prosthesis versus Cementless Implants

**DOI:** 10.3390/ma16041640

**Published:** 2023-02-16

**Authors:** Ahmed Abu-Awwad, Cristina Tudoran, Jenel Marian Patrascu, Cosmin Faur, Mariana Tudoran, Gabriel Mihai Mekeres, Simona-Alina Abu-Awwad, Andrei Nicolae Csep

**Affiliations:** 1 Department XV—Discipline of Orthopedics—Traumatology, “Victor Babes” University of Medicine and Pharmacy, Eftimie Murgu Square, No. 2, 300041 Timisoara, Romania; 2 “Pius Brinzeu” Emergency Clinical County Hospital, Bld Liviu Rebreanu, No. 156, 300723 Timisoara, Romania; 3 Department VII, Internal Medicine II, Discipline of Cardiology, “Victor Babes” University of Medicine and Pharmacy, Eftimie Murgu Square, No. 2, 300041 Timisoara, Romania; 4 Center of Molecular Research in Nephrology and Vascular Disease, Faculty of Medicine, University of Medicine and Pharmacy “Victor Babes” Timisoara, E. Murgu Square, Nr. 2, 300041 Timisoara, Romania; 5 Academy of Romanian Scientists, Ilfov Str. Nr. 3, 030167 Bucuresti, Romania; 6 Faculty of Medicine and Pharmacy, University of Oradea, Universitatii Street No.1, 410087 Oradea, Romania; 7 Doctoral School, “Victor Babes” University of Medicine and Pharmacy, Eftimie Murgu Square, No. 2, 300041 Timisoara, Romania

**Keywords:** cemented hip prosthesis, cementless hip prosthesis, polymeric cements, public health, total hip arthroplasty

## Abstract

(1) Background: Total hip arthroplasty (THA) is one of the most common procedures used for adult hip reconstruction, employing mainly two types of prostheses: cemented (CHP) and cementless (CLHP). This study aims to analyze the impact of the COVID-19 pandemic on THA with CHP and CLHP, in terms of the benefit/cost ratio. (2) Methods: This article represents a retrospective analysis of the differences concerning the benefit/cost ratio between THA with the two types of prostheses in 2950 patients admitted for THA in the two orthopedic clinics of our hospital between 1 January 2015–1 March 2020 in comparison with 1005 THA subjects seen between 1 April 2020–31 December 2022. (3) Results: In the first period, THA with CHP was performed in 45.83% of cases, while CLHP was used in 54.16% of patients. During the COVID-19 period, CHP was inserted in 52% of THA patients, while the other 48% had CLHP inserted, with a hospitalization duration reduced by over 50% for both types of implants (*p* ˂ 0.001). (4) Conclusions: CHP offered good outcomes, with quicker mobilization, and shorter hospitalization duration, compared to CLHP, but optimization of the patients’ management can be achieved mainly by reducing the length of hospitalization through an appropriate preoperative patient evaluation through a multidisciplinary approach, an aspect that was proven during the COVID-19 pandemic.

## 1. Introduction

Nowadays, in parallel with the aging population, osteoarthritis (OA) represents a tangible global public health problem. It represents one of the main reasons for disability in the elderly, with estimates that over 80% of individuals older than 65 years and 85% of those aged over 80, compared to 7% of adults under the age of 52, suffer from OA [1,2,3]. Worldwide, OA represents a disease that most quickly leads to a motor disability, a pathology that nevertheless affects the quality of life and life expectancy [4,5,6], especially in people already suffering from cardiovascular or metabolic diseases [7,8,9]. It is important to note that, given the high levels of obesity across Europe, an alarming increase in people living with disability due to OA is expected. Specifically, OA has become the fourth leading cause of disability worldwide by 2020, frequently associated with other chronic diseases [10,11,12]. As a consequence, joint replacement has emerged as a surgical technique to recover and preserve the mobility of these patients, as well as to improve their quality of life [6,13,14]. The incidence of total hip arthroplasty (THA) rose two–three times in the last decade [6,15]. 

THA has evolved as a result of improvements in the design of the femoral head prosthesis, the need to restore the acetabulum, the development of superior materials, but also the development of advanced surgical techniques, which have led to a better understanding of the mechanics of the hip [6,16,17]. Depending on the type of fixation, hip implants can be divided into cemented (CHP), non-cemented (CLHP), or a combination of the two methods—hybrid devices (HHP). Cemented THA uses polymethylmethacrylate (PMMA) as a binder that fixes the prosthesis in the bone cavity. Uncemented devices rely on the biological fixation of the prosthesis surface. The superiority of one type over another represents a topic of debate, each option being successfully used in THA worldwide, with some differences between geographical regions [17,18,19,20]. Of course, each type of prosthesis offers advantages and limitations. The most significant benefit of CHP is its lower cost, compared to CLHP ones, as well as the possibility of being used in patients with osteoporosis and poor bone structure or with a lack of bone stock, where biological fixation is difficult [17,21,22]. The main disadvantages of CHP are related to the possibility of degradation over time, leading to implant loosening, with the need of revision [19,23]. The cement is also associated with the risk of inflammation or even infection of the soft tissue adjacent to the implant site [24]. In past decades, many surgeons preferred CLHP, claiming increased durability over time and necessitating a shorter operating time, but the use of modern acrylic cement in THA offers a lower rate of complications and good durability, as described by certain recent meta-analyses [6,17,21]. Cement fixation, using a polyethylene cup and a standard-sized head, offers good outcomes, with the lowest risks and at the lowest costs [20]. The incidence of THA varies largely worldwide, depending on various national public health policies and their sanitary and economical status, because the costs of this procedure are rather high, although the long-term benefits are obvious [25]. According to Fordham et al., treatment costs for a primary THA were just over GBP 5000, but, compared with the implications of ‘no surgery’, the overall costs were lower [26,27]. Unexpected challenges for the progress of THP emerged during the COVID-19 pandemic, when health systems were overwhelmed worldwide and priority was given to hospitals treating infectious and pulmonary diseases, as well as to emergency services and intensive care units [28]. Many other hospitals, such as in the case of one of our orthopedic wards, have been completely converted to COVID-19 units to deal with the challenges represented by the increasing number of infected patients, and most of the remaining services had to reorganize their spaces and medical personnel, most of which were redirected to treat the SARS-CoV-2 infected patients. [29,30]. Therefore, economic resources were redistributed, mostly for protective equipment necessary for the medical personnel and specific therapies for COVID-19 patients, while elective surgical procedures, such as THA, were severely impacted. As a consequence, patients were drastically selected and asked to submit, before admission for THA, all the required laboratory assessments and interdisciplinary consults, with the associated therapy adjustments, especially anticoagulant and antiaggregant drugs [31]. 

This article brings new knowledge about the benefits offered by CHP and CLHP, aiming to demonstrate that hospitalization times, the shorter ones being optimal, are not in accordance with the type of chosen prosthesis. Due to innovative improvements in cement’s properties, CHP offers a proper and durable fixation, allowing a fast mobilization of the patient with a low rate of complications. This paper also aims to retrospectively analyze peculiar aspects related to costs and hospitalization duration of patients who underwent THA for hip OA, both with CHP and CLHP, in the orthopedic clinics of our hospital in the five years preceding the outbreak of the COVID-19 pandemic, in comparison to the following three years when, due to multiple restrictions, the hospitalization period had to be limited, a beneficial consequence that should be maintained and further improved.

## 2. Materials and Methods

### 2.1. Materials: Characteristics of the Cemented Prosthesis

Surgical cements have been used successfully for over half a century. Two types of bone cements can be distinguished: phosphocalcic cement (PCC) and acrylic ones [24,32].

The CHP is fixated in the acetabular cavity aided by orthopedic cement, most commonly, acrylic cement. Bone cements are supplied as powders and a liquid. The powder contains cement, an acrylic polymer mixed with an initiator (di-benzoyl peroxide), a radiopaque substance (zirconium oxide or barium sulfate), and an antibiotic. The liquid contains the monomer, a stabilizer (hydroquinone) to prevent premature polymerization, and an activator (dimethyl-para-toluidine) to favor polymerization at room temperature. When the powder polymer and liquid monomer are mixed, polymerization occurs.

**Figure 1:**
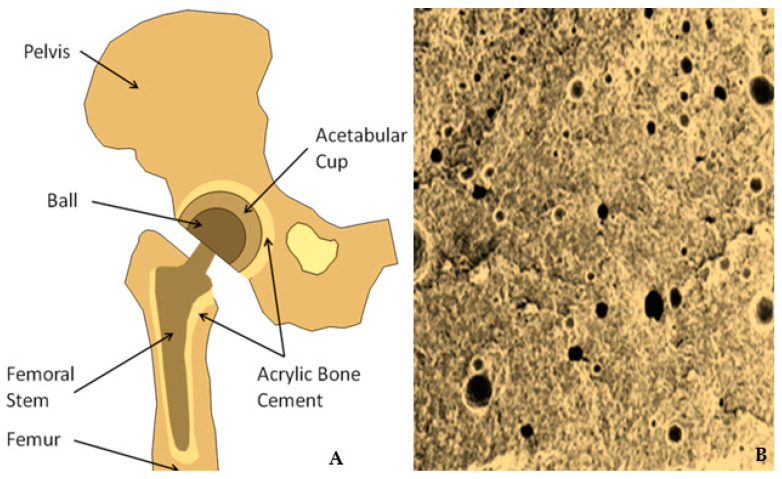
Schematic representation of CHP (**A**) and the cement’s electron microscopic aspect (**B**).

**Figure 2:**
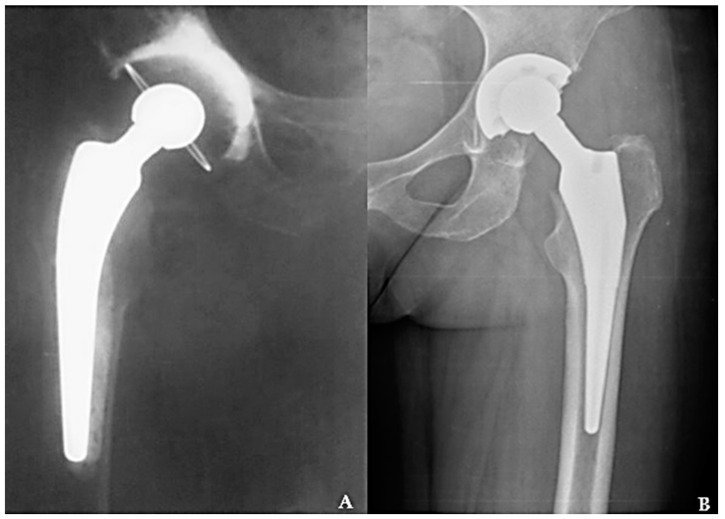
Total hip arthroplasty with CHP (**A**), versus a CLHP (**B**).

In orthopedic surgery, CHP is recommended mostly in elderly patients with associated comorbidities who suffer from osteoporosis in an advanced stage [19,20]. Due to the cementation, these prostheses have the advantage that they attach very well to the bone from the beginning (primary fixation) and allow full weight support on the treated leg, even from the first day after the prosthetic operation. The schematic representation of a CHP and the aspect of solidified polymeric cement in electronic microscopy are illustrated in Figure 1, and its radiographic aspect in Figure 2A, in comparison to a CLHP (B).

In contrast, CLHP has a porous surface, due to a coating with hydroxyapatite, is press-fitted in the bone surface, and, in time, the new bone will encase them. 

PCCs were created in 1979–1982, and, ever since, different PCC compositions have been studied and are commercially available due to their mechanical proprieties, comparable with other types of cement [33]. PCCs are obtained by a chemical reaction between two phases—a solid and a liquid one. The solid phase comprises one or more calcium phosphate compounds. The liquid phase is an aqueous solution that may contain calcium, phosphate, and various compounds, such as alginate and hyaluronate. Currently, despite the many formulations of PCC, there are only two possible end products for the PCC reaction: “brushite” (dicalcium phosphate dihydrate) and apatite, such as calcium-deficient hydroxyapatite or hydroxyapatite. PCCs are ionic types of cement used as bone substitutes because they easily adapt to bone defects. However, some inflammatory reactions have been reported when using too much cement.

The best-known bone cement types are presented in Table 1. Polymethylmethacrylate (PMMA) bone cements have progressed during the last 50 years to the most recent structures with nanoparticle additives, which are the most commonly used. Other types of known bone cement are PCCs and glass polyalkenoate cements (GPC) [32]. PCCs are bioresorbable and biocompatible, but are mainly used in cranial and maxillo-facial surgeries because of their low mechanical strength [24]. New additives have been developed for modern types of bone cement to overcome problems related to the loosening of the prosthesis, inflammatory reactions, or severe post-operative infection. Unfortunately, excepting imaging techniques, there are no other methods available to foresee the first degradation signs of the cement, as applicable for other cements [34,35]. Additives, such as vitamin E (<15%), small quantities of antibiotics (<5 g), and, more recently, silver nanoparticles, improve the quality of the prosthetic material and significantly reduce infection and inflammatory rates, while preserving the mechanical strength. Future work on PMMA bone cement should focus on testing various associations of additives to improve favorable properties.

The first cement containing antibiotics was used in the 1970s for the fixation of total hip arthroplasties, as anti-infectious prophylaxis. The purpose of these types of cement is to treat the infection locally by diffusing the antibiotic from the cement. It is a prophylactic antibiotic therapy, which is one of the means of reaching the germs that are at the cement–bone or cement–prosthesis interface. Bone cement can, thus, function as a local antibiotic-release matrix. Due to the high local antibiotic concentration in the implant environment, the use of bone cements has advantages over systemic antibiotic therapy [24]. When an implant encounters the tissue, the first and most rapid phase is the adsorption of proteins to the surface of the material. These are the proteins in the plasma that will adsorb based on their affinity for the surface. These proteins mediate the attachment of both surrounding tissue cells and pathogenic bacteria, especially Gram-positive ones [24]. As already known, tricalcium phosphate (TCP) confers more interesting properties on cement than alumina, in particular from the point of view of biology and degradability. 

For less than 20 years, some CLHPs have been coated with hydroxyapatite. Hydroxyapatite is the mineral form of calcium in bone. This facilitates osteointegration of the prosthesis in the short term. In the long term, the results seem identical to a conventional cementless rod.

Placing a CLHP requires a certain practice and expertise of the surgeon, because one must feel the quality of the bone and find the size that fits best. This is probably one reason why we find right and left dentures without cement. The tribological behavior of hip prostheses derives from the biomechanics of this joint. The normal walking cycle lasts about a second and shows a significant peak of force during heel-to-ground contact. When the hip is replaced by a prosthesis, during the swinging phase of the leg, a small separation between the head and the cup may occur. This separation is on the order of a millimeter and is called decapitation. During the contact of the heel with the ground, due to this decapitation, a shock occurs between the head and the cup, with a force that can reach up to 9 times the body weight and lasts about ten milliseconds. This friction force may determine a more rapid degradation of the two metal parts of the prosthesis. To prolong the lifespan of hip implants, Jamari et al. developed a texturing technique of the intra-articular bearing surfaces with bottom dimples and tested them by using 3D geometrical models that mimicked the physiological loading of the hip [36].

### 2.2. Patients Groups

The paper represents a retrospective analysis of the differences regarding the duration of hospitalization, benefits, complications, and expenditure costs of THA performed either with CHP or CLHP, in patients diagnosed with OA of the hip, and admitted either between 1 January 2015–1 March 2020 or between 1 April 2020–31 December 2022 in both Orthopedic Departments of the “Pius Brînzeu” Emergency County Clinical Hospital Timisoara. We analyzed these data by using the hospital’s electronic database and its information storage method, more specifically the Info World—Hospital application. Filtering was performed using the WHO ICD-10 diagnostic codes of interest, namely M16.1, M16.0—other primary coxarthrosis, used for both admission and discharge diagnoses. We identified a total of 2950 cases with hip OA, with a mean age of 61.5 ± 13 years, who underwent THA during the period 1 January 2015–1 March 2020, and another 1005 patients admitted for the same reasons between 1 April 2020–31 December 2022, with a mean age of 58.4 ± 11 years. We only included in our study patients who had a favorable outcome, with postoperative complications that have been successfully treated.

The prostheses that were available during the study period were from Stryker- Portage, MI, USA: Trident hemispherical shell, hemispherical acetabular shell, and Arc-deposited CPTi (commercially pure titanium), available with or without PureFix HA (hydroxyapatite) coating, and compatible with Trident Torx acetabular screws, sizes: 42–74 mm, solidback and clusterhole option, clusterhole options: 42–50 mm with 3 holes (52–74 mm) or 5 holes were employed. Another type was Accolade II, where a complex relationship between stem length and implant stability exists, and the shortening of the stem length without geometry optimization has been shown to increase the potential for micromotion, which is considered a strong indicator for implant failure. Accolade II utilizes the SOMA database and stability analyses to establish an optimized length for each stem size, which not only accommodates muscle-sparing approaches but also demonstrated improved initial stability. In the case of CHP, only acrylic cement was utilized, and the employed cementing technique was the one recommended in the manufacturer instructions. We used a special mixer to mix the cement with the provided ampoules. After mixing the cement for 2–3 min, and after obtaining the optimal viscosity, we inserted it into the femoral canal, and to avoid migration, we used the pressurization technique for the femoral component. We also employed this technique for the acetabular cavity. The patient data were analyzed according to various factors, such as hospitalization duration, with a focus on the pre- and post-surgery period duration and expenditure, depending on the type of implanted hip prosthesis: cemented versus non-cemented. 

We utilized the following inclusion criteria: patients aged between 45 and 90 years, able to understand and sign the informed consent form, without previous trauma to the respective hip, good biological status, good functional status, normal movements from a kinetotherapeutic point of view, as far as locomotor possibilities are concerned, because of hip arthrosis.

The exclusion criteria consisted of patients with hip OA due to repeated trauma to the hip, the current admission being, practically, for the second or third surgical intervention at the respective level, aged younger than 45 years and over 90 years, patients with multiple associated pathologies that determine a precarious biological status, and patients who did not walk before the development of arthrosis due to neurological causes.

### 2.3. Data Analysis

Data analysis was performed using SPSS v.25.0 (Statistical Package for the Social Sciences, Chicago, IL, USA) for Linux Mint 19. Continuous variables were presented as a mean and standard deviation (SD) or median and associated quartiles (Q1-25 percentage quartile, Q3-75 percentage quartile), and categorical data were presented as counts (percentages). The bias-corrected and accelerated (BCa) bootstrap interval (1000 bootstrap samples) was used to calculate the 95% confidence interval. We performed descriptive and inferential statistical analysis to summarize the characteristics of the study population. The results of the Shapiro–Wilk normality test showed a non-Gaussian distribution, which is why we continued to use nonparametric tests. To evaluate the differences related to the costs of the prosthesis in groups, we applied the chi-squared test (χ2) and Fisher exact test (Freeman–Halton extension). 

A *p*-value of less than 0.05 was considered to indicate statistical significance. 

All of the patient data were anonymized, and the research ethics committee and the institutional review board of our university approved this study (042/10.12.2018). The study was performed following the World Medical Association Declaration of Helsinki (revised in 2000, Edinburgh). All patients provided written informed consent before study entry. In view of hospital admission, all patients signed the standardized informed consent form required by the national health authorities, by which they consented to their data being used for medical research and education purposes.

## 3. Results 

Our study included two main subsets of patients selected from the “Pius Brînzeu” Emergency Clinical County Hospital Timișoara database. The first category was represented by 2950 subjects with OA of the hip, scheduled for THA, who were admitted during the 5-year period, between 1 January 2015–1 February 2020 in the orthopedic departments of our hospital. In 1352 cases (45.83%), THA with CHP was performed, while in 1598 subjects (54.16%) a CLHP was inserted. Regarding the structure of our study group, there were 885 males (30%) and 2065 women (70%) with ages between 48 and 86 years and a mean age of 61.5 ± 13 years. Demographic data, hospitalization duration, and expenditure, as well as the value of the implanted prosthesis and the necessary materials, are presented in Table 2.

Most patients had a favorable evolution, with an average inpatient duration of 8 days in the case of CHP, and 11 days for CLHP, with a maximum of 14 days inpatient admission in some cases. Thus, we noticed a higher postoperative complication rate compared to usual norms with most of them occurring in the first 24 h post-surgery and consisting of cardiovascular, pulmonary, and neurological complications. In terms of the duration of hospitalization, this was determined by two main components: (1) the pre-surgical period, which depended on several factors: the urgency degree of the intervention, the clinical status of the patient, associated diseases and chronic therapies, the number of pre-operative investigations required, and the preoperative preparation; (2) the post-operative period was determined mainly by the evolution and recovery of the patient and the number of required days for remobilization. Concerning hospitalization costs, this consisted of variable aggregated expenses, determined by the number of hospitalization days multiplied by the accommodation and medication costs/day (around EUR 124). The expenses for associated laboratory tests and other investigations (e.g., radiographs or osteodensitometry investigations) were calculated at an approximate total amount of about EUR 70. The value of a CHP, including all necessary materials and maneuvers, is around EUR 905. However, costs change if the same patient needs a CLHP due to biological eligibility and the quality of the bone material. The value of this type of prosthesis, including all materials used, was circa EUR 1500, as described in Table 2.

To more accurately highlight the differences regarding the management and expenses for each type of prosthesis, we detailed the duration of hospitalization/expenses in these two situations. 

In the first situation, we analyzed the duration of hospitalization/expenditure breakdown for patients diagnosed with OA of the hip, where a THA with CHP was performed, and the median duration of hospitalization was 8 days, comprising an average of 3 days for preoperative inpatient and 5 days postoperative recovery stay. The total expenditure allocated for each patient was composed of fixed expenses (the price of cemented hip prosthesis and necessary materials = EUR 905, the value of laboratory tests and other investigations of about EUR 70) and variable expenses, depending on the number of days of hospitalization (related to the value of accommodation and meals and medication during hospitalization, of about EUR 998), leading to a total cost of EUR 1972 for an average inpatient admission of 8 days. 

In the second example, we analyzed the same parameters for patients diagnosed with OA, where the therapeutic decision for THA with CLHP was taken, and the postoperative hospitalization time was 11 days. Thus, the fixed costs (the price of the uncemented hip prosthesis and necessary materials were approximately EUR 1500, while laboratory tests and other investigations cost EUR 70) and the expenses related to hospitalization and medication administered during hospitalization were EUR 1372, amounting to a total of EUR 2941 for 11 days of hospitalization.

During the COVID-19 pandemic and the following period, a total number of 1005 patients were admitted for THA in our hospital. They were aged between 45 and 90 years, with a mean age of 58.4 ± 11 years. As for their gender distribution, there were 712 female patients (70.84%) and 293 male patients (29.15%), thus similar to the first subset of patients. In 522.6 cases (52%), THA was performed with CHP, while in 482.4 cases (48%), a CLHP device was inserted. The average hospital stay was 3 days for CHP and 5 days for CLHP, as shown in Table 3. The hospitalization period was shorter since, during the COVID-19 pandemic, the Romanian Ministry of Health introduced a series of restrictions, so that patients were discharged faster than in the pre-COVID-19 period, even after a THA. Moreover, one of the orthopedic clinics and its medical personnel were redirected to a COVID-19 dedicated unit during various periods, covering almost two years. It should be mentioned that, in view of admission for the scheduled THA, all patients were asked to present a negative PCR test, performed within 48 h, and a set of laboratory analyses necessary for the intervention, collected and validated in advance. All these changes helped us to optimize the hospitalization time, thus regulating the expenses for each patient.

As a consequence of the experience gained during the COVID-19 period, hospitalization time has been further shortened, with patients being discharged after either 3 days in the case of CHP or 5 days in the case of CLHP.

**Figure 3:**
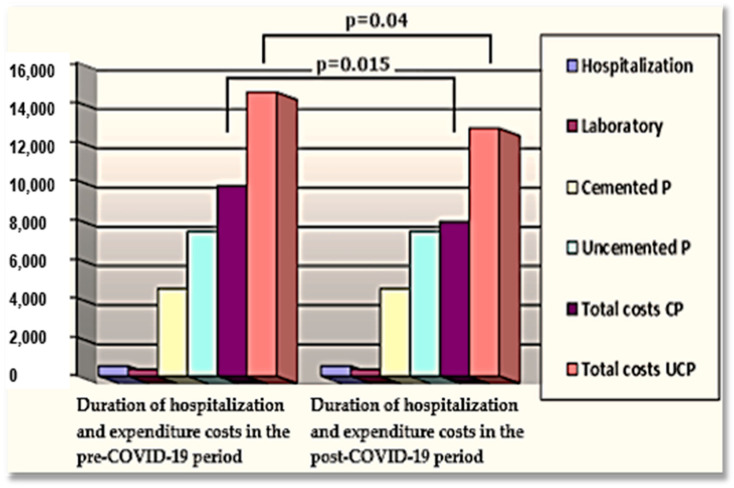
Data concerning the management of THA with CHP and CLHP preceding and following the COVID-19 pandemic. Legend: prosthesis—P; cemented prosthesis—CP; uncemented prosthesis—UCP.

In comparison with the first situation, where we presented the expenditure account of a patient diagnosed with degenerative OA of the hip, where the therapeutic decision was of a THA with CHP, during the COVID-19 pandemic and in the following period, we were able to reduce the preoperative hospitalization time by using an optimized coordination in terms of time and engagement with the internal medicine/cardiology teams, who performed a preoperative evaluation of all patients and made the necessary therapeutic adjustments, so that patients scheduled for THA arrived at the hospital in optimal conditions for anesthesia and surgery, from a medical and biological point of view. Thus, the average duration of hospitalization was reduced from 8 to 5 days, which involved an adjustment of the expenditure to EUR 1598 for an inpatient stay (see Figure 3).

In the second case, we analyzed the management of a patient diagnosed with hip OA, where the therapeutic decision involved a THA with CLHP. After applying the same management strategy, with a proper preoperative evaluation and therapy of any associated pathologies, leading to the patient presenting in their best biological status prior to surgery, the former hospitalization duration of 11 days was reduced to 5 days, and the allocated expenses were decreased, from EUR 2941 to EUR 2567 (12.72%), see Figure 3.

When carefully analyzing the group of patients included in our study from the point of view of prostheses, we can confirm that patients who underwent a CHP surgery had, in comparison to those who benefited from a CLHP, a shorter recovery time and a more favorable evolution, from a kinetic point of view, which allowed for an earlier discharge than in the case of those with a CLHP. 

## 4. Discussions

Internationally, degenerative OA of the hip is a constantly growing pathology, owing to an increase in life expectancy, and, therefore, it is expected that the number of cases will rise in line with the advancing age of the global population. The rapid growth of the number of joint replacement procedures, mainly THA and total knee arthroplasty, in past decades was analyzed by researchers from Poland in a recent study, and they advanced an augmentation of the number of THA from 34% in 2020 to 284% in 2040, which would generate significantly higher costs and an increasing risk of complications, often requiring repeated surgical procedures [37]. Considering that OA is a disease more frequently occurring with advanced age, many of these patients have comorbidities, especially cardiovascular and metabolic diseases, and are currently treated with medication that may determine coagulation issues [5,7,31,38]. In terms of gender distribution, our study indicated that mostly women (70%) suffered from degenerative OA of the hip, many of them also suffering from associated osteoarticular pathologies, especially osteoporosis that can influence the surgical method and negatively impact their prognosis and recovery, thus prolonging the postoperative period [11,13].

Data from meta-analyses [17,23] of large studies from national registries from the UK, Sweden, USA, and New Zealand on the topic of THA report excellent outcomes, with low revision rates for CHP, concluding that cemented fixation is the golden standard in THA, in terms of re-intervention rates in all patients, regardless of age [13,39]. The review of Zhang et al. [13] analyzed the most recent annual reports from the international joint arthroplasty registries from Sweden, Norway, England—Wales, Australia, and New Zealand with more than five years of follow-up. They also reviewed the most significant randomized clinical trials and meta-analyses in the literature and concluded that in THA, CHP resulted in an overall better long-term outcome than the use of CLHP. As a general remark, CHP implantation resulted in better survival in older patients, while cementless fixation determined superior outcomes in younger patients but are also used with or without bone graft for revisions [19,40]. Surprisingly, during the COVID-19 pandemic, when elective surgical procedures, including THA, were postponed worldwide [41,42,43], the same tendency of implanting CHP more often than CLHP was maintained in our study (52% versus 48%). This aspect can be probably explained by the increased prevalence of patients with more advanced OA and associated osteoporosis, who could not postpone the THA any longer, the lower cost of CHP, and the possibility of a more precocious patient mobilization. Interestingly, after an initial dramatic decrease in THA numbers, of about 45% in our wards, due to sanitary restrictions, and after a reduction of beds during 2020, the hospitalization duration was reduced by almost 50%, allowing a new increase in THA procedures with comparable complication rates. Similar results were reported by several authors [37,44]. In their study, Gordon et al. analyzed 77,797 patients who underwent elective THA in 2019 (n = 43,667) and 2020 (n = 34,130) and noticed a 24.5% decline of this procedure [45]. Another study conducted on 17,039 patients admitted for joint replacement surgery in university and community hospitals in Taiwan revealed an even greater reduction, of about 60%, of these procedures during the COVID-19 pandemic [46]. A similar study from Poland reported a decrease of around 30% [47]. Many hospitals specialized in joint replacement surgery had to adapt and reorganize their activity, some of them reporting even results [41,48]. However, it appears that having had to repeatedly postpone surgical procedures due to multiple sanitary restrictions profoundly affected the mental health of OA patients [49,50]. 

The duration of hospitalization, expenditure costs, and management of surgical treatments differ largely, depending on the chosen treatment option. It is well known that the main advantage of CHP is its lower price when compared to CLHP, where the added porous metal coating necessary to achieve bone infiltration renders it more expensive. The first one provides a good fixation immediately after insertion and is well suited for patients with poor bone structure or bone loss; however, the second type offers a lower operative time [13,27,51]. 

In our study, in the first studied period, in the case of CHP, the total duration of hospitalization was 8 days, leading to an expenditure of around EUR 1972/patient, which was significantly lower than that required for CLHP, where the duration of hospitalization was 11 days and a total of EUR 2941/patient (*p* = 0.015), Table 2. These hospitalization durations were rather long because many patients had associated pathologies, often requiring adjustments of concomitant therapies, especially antiaggregants or anticoagulants, and needed multiple laboratory explorations, which were performed in the hospital. Considering that the cost of each type of implant and the necessary materials were identical, a reduction of the cost-efficiency ratio could be achieved only by reducing the hospitalization time. During the COVID-19 pandemic, and in the following period, we achieved a significant reduction of the hospitalization duration by better management, involving the patients being asked to perform the required evaluations in outpatient services. 

In most Western countries, a patient who underwent surgery for hip OA is discharged after approximately 3–5 days postoperatively (according to the literature data), depending on the individual’s biological status and comorbidities [4,19,22,51]; the inpatient stay was reduced further during the COVID-19 pandemic, some authors reporting even very low time-spans such as 1.8 days [45]. The cost of the intervention in various hospitals worldwide varies largely: in Belgium (Brussels) it is about USD 13,660/EUR 12,707, compared to US cities, where the price ranges between USD 30,000/EUR 27,908 and USD 112,000/EUR 104,190 [15,21,23,39,52,53]. Costs of surgical procedures performed in most foreign countries are frequently compared to costs in the USA, thus, it is important to interpret them with caution [13,15]. Regulations regarding the standards of infection control, sterilization, medical malpractice, and additional health care-related factors may differ significantly between countries, which can dramatically affect overall surgery costs [4,13,15,20,22].

The average period of hospitalization in most hospitals in Western Europe for patients with this pathology, treated using the two methods, in the absence of complications, is significantly lower, compared to the hospitalization duration in the “Pius Brînzeu” Emergency County Clinical Hospital Timisoara. If we consider how hospitals in Western Europe manage hospitalization days for patients with degenerative OA of the hip, treated surgically, reducing the number of hospitalization days for THA is imperative. With the reduction of inpatient stays, the hospitalization expenditure will automatically be lower, including costs for the administered medication. In this study, we demonstrated that the reduction in the number of days of hospitalization imposed by the COVID-19 pandemic resulted in a significant reduction of the cost/benefit ratio, both in the case of CLHP and CHP patients. Even if non-cemented prostheses are expensive from a financial point of view but hold the advantage of a more tolerable revision surgical procedure, we consider that cemented prostheses ensure a better quality of treatment, a higher quality of infection prevention (due to the cement being treated with antibiotic), as well as ensuring a faster postoperative mobilization for the patient, as the bone stock is fortified with the help of the cement used. Perpetual research studies are targeting a more rigorous testing of the prosthesis’ components under various mechanical stress conditions. In a recent study [54], the risk of wear for two categories of polyethylene liners, both for CHP and CLHP as well, was analyzed by finite element analysis in 72 simulations, and cemented solution seemed superior regarding the risk of wear, with respect to the polyethylene thickness (over 5 mm was recommended) [54]. Another direction is development of superior materials and improved cements to overcome the risks of infection and prosthesis degradation [36,37,55]. Innovative techniques, such as selective laser melting technology, allowed the production of complex porous structures, mimicking the natural structure of the human bone but preserving its mechanical properties at the same time, which could be used for the production of the hip prosthesis [56].

As a future direction, we intend to maintain the hospitalization period obtained during this period and even shorten it by a multidisciplinary approach of patients’ management. Another project is to carry out a pilot study by hospitalizing the patients only for 24 h after surgery and creating an integrative program involving multiple digital methods of communication for the ambulatory post-operative treatment and patients’ follow-up. A similar project was developed during the COVID-19 pandemic by Kamecka et al., who proposed the implementation of telemedicine tools for the post-hospital patient care proceedings after THA that should be carried out by interdisciplinary teams [44].

Our research has several limitations. The most important one is the rather small number of cases who underwent THA. This can be explained by the reality that our two departments are not dedicated joint replacement surgery settings, as they are also the main traumatology emergency wards for a wide geographical area. Another limitation results from the type of patients admitted in our departments, who often have multiple comorbidities, associated therapies, and/or social problems, who were not eligible for other, specialized joint replacement surgery wards. As mentioned before, the COVID-19 pandemic, with its ensuing restrictions, supplementarily impacted our activity.

## 5. Conclusions 

Our study indicated mostly women (70%) among the patients undergoing THA. Polymeric cemented implants appeared to offer good outcomes, with a quicker mobilization, and a lower hospitalization duration, compared to cementless devices, which are more appropriate for younger patients. Independent of the type of prosthesis employed for THA, an optimization of patient management could be achieved mainly by reducing the length of hospitalization through an appropriate preoperative evaluation and preparation of the patient by a multidisciplinary approach. The example was set by the inherent, innovative efforts during the COVID-19 pandemic, when we achieved a significant reduction of the hospitalization duration, by using better time and team management, and we intend to further improve our achievements by employing a multidisciplinary approach and digital communication methods.

## Figures and Tables

**Table 1:** Composition of the most common antibiotic-loaded acrylic cements used in orthopedic surgery.

	DePuy CMW			Stryker Simplex	Palacos R+G	COPAL	
	Heritage Cement						
Components	CMW1	CMW3	CMW End			G+V	G+C
Powder					40.8	42.85	42.85
Polymethylmethacrylate	84.73	83.88	65.53	14.83	33.8	35.41	35.41
Methylmethacrylate/methacrylate/styrene	-	-	19.7	74.13	-	-	-
Gentamicin sulfate	4.22	4.22	4.22	-	0.5	0.5	1
Colistimethate sodium	-	-	-	0.59	-	-	-
Tobramycin	-	-	-	1.23	-	-	-
Vancomycin	-	-	-	-	-	2	-
Clindamycine	-	-	-	-	-	-	1
Barium sulfate	9.1	10	9.75	9.88	-	-	-
Zirconium dioxide	-	-	-	-	6	4.27	4.27
Benzoyl peroxide	1.95	1.9	1.8	-	0.2	0.32	0.32
Liquid							
Methylmethacrylate	98.23	96.54	98.01	97.5	18.4	18.4	18.4
N,N-dimethyl-P-toluidine	0.81	2.49	1.98	2.4	0.4	0.4	0.4

**Table 2:** Characteristics of patients, duration of hospitalization, and expenditure of hip arthroplasty.

Characteristics	Cemented Hip Prosthesis	Cementless Hip Prosthesis	*p*
Number of patients	1352—45.83%	1598—54.16%	NS
Male/Female gender	M 300/F 1052	M 585/F 1013	-
Mean age (years)	68.8 ± 9.76	56.6 ± 12.22	0.032
Duration of hospitalization: - Days until surgery - Days after surgery	8 3 5	11 3 8	0.025 NS 0.025
Days until mobilization	1	3	0.01
Expenditure related to hospitalization/day (EURO) Value of prosthesis and necessary materials (EURO)	1067 905	1441 1500	0.012
Actual expenditure/hospitalization	1973	2940	0.015
Optimized expenditure/hospitalization	1598	2566	0.04

**Table 3:** Characteristics of patients, duration of hospitalization, and expenditure costs for THA during the COVID-19 period.

Characteristics	Cemented Hip Prosthesis	Cementless Hip Prosthesis	*p*
Number of patients	522.6—52%	482.4—(48%)	NS
Male/Female gender	M 212.2/F 310.4	M 187.2/F 295.2	-
Mean age (years)	69.7 ± 8.32	58.4 ± 11 years	0.035
Duration of hospitalization: - Days until surgery - Days after surgery	3 1 2	5 1 4	0.025 NS 0.025
Days until mobilization	1	1	0.01
Expenditure related to hospitalization/day (EURO) Value of prosthesis and necessary materials (EURO)	449 905	1067 1500	0.012
Actual expenditure/hospitalization	1354	2567	0.015

## Data Availability

Not applicable.
